# Supplementary material for: A Critical View of the Application of the APEX Software (Aqueous Photochemistry of Environmentally-Occurring Xenobiotics) to Predict Photoreaction Kinetics in Surface Freshwaters
Source: Molecules. 2019 Dec 18;25(1):9. doi: 10.3390/molecules25010009 (PMC7017383; doi:10.3390/molecules25010009)
Supplement: Supplementary file 1 [file molecules-25-00009-s001.zip › 3.2.4_gcc-4.4.0.zip!/3.2.4_gcc-4.4.0/3.2.4_gcc-4.4.0/doc/octave/html/interpreter/The-_003ccode_003eswitch_003c_002fcode_003e-Statement.html]

The @code{switch} Statement - Untitled


Next: The <code>while</code> Statement,
Previous: The <code>if</code> Statement,
Up: Statements


---

### 10.2 The `switch` Statement

It is very common to take different actions depending on the value of
one variable. This is possible using the `if` statement in the
following way

```
     if (X == 1)
       do_something ();
     elseif (X == 2)
       do_something_else ();
     else
       do_something_completely_different ();
     endif
```

This kind of code can however be very cumbersome to both write and
maintain. To overcome this problem Octave supports the `switch`
statement. Using this statement, the above example becomes

```
     switch (X)
       case 1
         do_something ();
       case 2
         do_something_else ();
       otherwise
         do_something_completely_different ();
     endswitch
```

This code makes the repetitive structure of the problem more explicit,
making the code easier to read, and hence maintain. Also, if the
variable `X` should change its name, only one line would need
changing compared to one line per case when `if` statements are
used.

The general form of the `switch` statement is

```
     switch expression
       case label
         command_list
       case label
         command_list
       ...
     
       otherwise
         command_list
     endswitch
```

where label can be any expression. However, duplicate
label values are not detected, and only the command\_list
corresponding to the first match will be executed. For the
`switch` statement to be meaningful at least one
`case` label command\_list clause must be present,
while the `otherwise` command\_list clause is optional.

If label is a cell array the corresponding command\_list
is executed if *any* of the elements of the cell array match
expression. As an example, the following program will print
Variable is either 6 or 7.

```
     A = 7;
     switch A
       case { 6, 7 }
         printf ("variable is either 6 or 7\n");
       otherwise
         printf ("variable is neither 6 nor 7\n");
     endswitch
```

As with all other specific `end` keywords, `endswitch` may be
replaced by `end`, but you can get better diagnostics if you use
the specific forms.

One advantage of using the `switch` statement compared to using
`if` statements is that the labels can be strings. If an
`if` statement is used it is *not* possible to write

```
     if (X == "a string") # This is NOT valid
```

since a character-to-character comparison between `X` and the
string will be made instead of evaluating if the strings are equal.
This special-case is handled by the `switch` statement, and it
is possible to write programs that look like this

```
     switch (X)
       case "a string"
         do_something
       ...
     endswitch
```

- Notes for the C programmer
